# Interoception in obsessive-compulsive and major depressive disorders

**DOI:** 10.3389/fpsyg.2026.1856945

**Published:** 2026-08-05

**Authors:** Nicolette Recchia, Goi Khia Eng, Jeanmarie Harvey, Rachael Moldow, Katherine A. Collins, Emily R. Stern

**Affiliations:** 1 Department of Psychiatry, New York University Grossman School of Medicine, New York, NY, United States; 2 Clinical Research Division, Nathan S. Kline Institute for Psychiatric Research, New York, NY, United States; 3 Neuroscience Institute, New York University Grossman School of Medicine, New York, NY, United States

**Keywords:** heartbeat counting, interoception, interoceptive accuracy, interoceptive sensibility, major depressive disorder, obsessive-compulsive disorder

## Abstract

Atypical interoception is a transdiagnostic feature of psychiatric illness. Interoceptive alterations have been demonstrated in both obsessive-compulsive disorder (OCD) and major depressive disorder (MDD). However, the nature and direction of differences between patients and controls have been inconsistent, and interoception has not been directly compared between OCD and MDD in the same study. The current study addressed this gap by comparing OCD patients, MDD patients, and healthy controls (HCs) on three different facets of interoception: accuracy (IAcc; objective ability to detect bodily sensations), confidence in interoceptive ability (self-reported ability to detect bodily sensations accurately), and one dimension of interoceptive sensibility (IS; self-reported tendency to notice bodily sensation). Measures of IAcc and confidence were derived from responses in a heartbeat tracking task, whereas IS was measured with the Multidimensional Assessment of Interoceptive Awareness (MAIA) Noticing subscale. There were no differences between the groups in IAcc. Individuals with OCD displayed lower confidence in their interoceptive ability relative to both HC and MDD, although the pairwise effects did not survive multiple comparison-correction. Lower confidence was also nominally associated with greater obsessive-compulsive symptom severity. Both OCD patients and MDD patients exhibited significantly elevated IS compared to HCs, however, IS did not differ between patient groups. Exploratory analyses suggested a positive association between IAcc and confidence in interoceptive ability within OCD patients, while no associations between IAcc and confidence were found in MDD or HCs. These findings provide preliminary evidence for shared and potentially unique alterations of interoception in OCD and MDD.

## 1 Introduction

Interoception, defined broadly as the detection, integration, and interpretation of bodily signals, is fundamental to the regulation of physiological states, emotions, and self-representations (Craig, 2002). Three distinct facets of interoception have been proposed: interoceptive accuracy, interoceptive sensibility, and interoceptive awareness (Garfinkel et al., 2015). Interoceptive accuracy (IAcc) is the ability to accurately identify internal bodily signals and can be measured with behavioral tasks requiring an individual to detect bodily sensation where these sensations (or the signals generating them) can be objectively quantified (e.g., counting heartbeats). Interoceptive sensibility (IS) is a multifaceted construct that broadly refers to self-reported beliefs related to bodily sensations, including the tendency to notice, attend to, interpret, and regulate responses to bodily signals. IS is typically measured using self-report questionnaires [e.g., the Multidimensional Assessment of Interoceptive Awareness (MAIA, Mehling et al., 2012)] or self-reported confidence in performance during an IAcc task (Garfinkel et al., 2015). Interoceptive awareness is the metacognitive awareness of interoceptive ability, operationalized as the correspondence between objective (IAcc) and self-perceived accuracy (i.e., confidence) on an IAcc task. More recently, Murphy et al. (2019) proposed a 2 * 2 framework that further distinguishes interoceptive measures in terms of what they assess (accuracy in detecting sensation vs attention given to sensation) and how they are implemented (objective performance vs. self-report).

Aberrant interoception is a transdiagnostic feature of psychiatric illness (Khalsa et al., 2018) and theoretical accounts suggest that it may be central to both obsessive-compulsive disorder (OCD) (Dar et al., 2021) and major depressive disorder (MDD) (Harshaw, 2015). In individuals with OCD, aversive bodily sensations often precede repetitive behaviors (Bragdon et al., 2021; Ferrao et al., 2012; Wilson et al., 2025) while somatic disturbances are commonly reported by individuals with MDD (Eggart et al., 2019; Vaccarino et al., 2008), highlighting altered IS as a clinically relevant feature of both disorders.

Empirical studies have reported alterations of IAcc, confidence in interoceptive ability, and IS, including the self-reported tendency to notice bodily sensations, in OCD groups (Bragdon et al., 2021; Demartini et al., 2021; Eng et al., 2020; Schultchen et al., 2019; Yoris et al., 2017) and MDD groups (Dunne et al., 2021; Furman et al., 2013; Karanassios et al., 2021; Schulz et al., 2022; Zhou et al., 2024) when separately compared to control groups, however, the nature and direction of interoceptive differences have been inconsistent. A recent study found reduced IAcc and lower self-reported confidence in interoceptive ability, but similar levels of IS, in a transdiagnostic patient sample (consisting of individuals with OCD, MDD, anxiety disorders, and schizophrenia spectrum disorders) compared to healthy controls (HCs) (Critchley et al., 2023). Significant differences between patient groups were primarily driven by lower IAcc and IS in individuals with schizophrenia spectrum disorders (Critchley et al., 2023).

Although Critchley et al. (2023) included OCD and MDD samples in their pooled analyses, the OCD sample size was small (*n* = 9), which limited their direct comparison to other diagnostic groups. To our knowledge, no prior study has examined interoception in OCD and MDD patients within the same study using identical measures. A direct comparison between patient groups may aid in the identification of common- and disorder-specific interoceptive profiles. The current study addressed this gap by comparing OCD, MDD, and HCs on IAcc, confidence in interoceptive ability, and IS (specifically the tendency to notice bodily sensation) during a heartbeat tracking task, with the hypothesis that both patient groups would differ from HCs but be similar to each other. Additionally, an exploratory aim was to examine relationships among interoceptive measures and between interoception and symptom severity within each group.

## 2 Methods

### 2.1 Participants

Participants were recruited at Nathan Kline Institute for Psychiatric Research and New York University Grossman School of Medicine between September 2021 and December 2023 as part of two larger projects. The study was approved by Institutional Review Boards at each location and all subjects provided written informed consent. The final sample consisted of 41 individuals with OCD, 38 individuals with MDD, and 35 HCs.

A trained rater made all diagnoses based on DSM-5 criteria using modules from the Structured Clinical Interview for DSM-5 (SCID) (First et al., 2015) to diagnose OCD and MDD, and the Mini-International Neuropsychiatric Interview (MINI) (Sheehan et al., 1998) for all other psychiatric conditions. Individuals in the OCD group had a diagnosis of OCD without a comorbid diagnosis of MDD and individuals in the MDD group had a diagnosis of MDD without a comorbid diagnosis of OCD. Patients were excluded for lifetime presence of schizophrenia spectrum or bipolar disorder, or current severe alcohol or substance use disorders. HCs were free of psychiatric diagnoses and psychotropic medications. Information regarding DSM-5 comorbidities and psychotropic medications are described in the Supplement: 1.1, Supplementary Table 1, 2.

### 2.2 Clinical interviews

The Yale-Brown Obsessive Compulsive Scale (Y-BOCS) (Goodman et al., 1989) and Inventory of Depressive Symptomatology (IDS) (Rush et al., 1986) assessed disorder-specific symptom severity.

### 2.3 Interoceptive measures

#### 2.3.1 Interoceptive accuracy and confidence in interoceptive ability

A heartbeat tracking task (HTT) (Schandry, 1981) was used to assess both IAcc and confidence in interoceptive ability. This task is widely used in the literature (Critchley et al., 2023; Demartini et al., 2021; Garfinkel et al., 2015; Schultchen et al., 2019), facilitating comparisons with past work. In the HTT, participants are asked to silently count their heartbeats during each of 6 intervals of varying length (3 intervals presented twice: 25s x2, 35s x2, and 45s x2) presented in a pseudorandom order where intervals of the same length did not occur consecutively. Auditory beeps signaled the start and end of each interval (Supplement: 1.2). Cardiac waveforms were recorded using a pulse transducer placed on the right index finger, and participants were instructed not to use their fingers to check their pulse during the task.

After each interval, participants verbally reported the number of heartbeats they counted. Participants were required to keep their eyes closed during counting to limit distraction. Consistent with other studies using the task (Demartini et al., 2021; Karanassios et al., 2021; Schultchen et al., 2019), IAcc was derived using the following formula:


$$I\,n\,t\,e\,r\,o\,c\,e\,p\,t\,i\,v\,e\,A\,c\,c\,u\,r\,a\,c\,y=\frac{1}{6}\,\sum \left(1-\frac{\left|A\,c\,t\,u\,a\,l\,n\,u\,m\,b\,e\,r\,o\,f\,h\,e\,a\,r\,t\,b\,e\,a\,t\,s-R\,e\,p\,o\,r\,t\,e\,d\,n\,u\,m\,e\,r\,o\,f\,h\,e\,a\,r\,t\,b\,e\,a\,t\,s\right|}{A\,c\,t\,u\,a\,l\,n\,u\,m\,b\,e\,r\,o\,f\,h\,e\,a\,r\,t\,b\,e\,a\,t\,s}\right)$$

IAcc scores range from 0 to 1, with higher scores representing greater accuracy.

In addition to counting heartbeats, participants rated their confidence in the accuracy of their counting (from 1[not at all confident]–9[completely confident]). Average confidence scores reported across the 6 time-intervals provided an overall measure of participants’ confidence in interoceptive ability.

#### 2.3.2 Interoceptive sensibility

The Multidimensional Assessment of Interoceptive Awareness (MAIA) (Mehling et al., 2012) is a 32-item self-report scale assessing different dimensions of IS with 8 different subscales. The current study focused only on the Noticing subscale, which assesses the general tendency to notice and be aware of bodily sensations (e.g., “I notice changes in my breathing, such as whether it slows down or speeds up”), consistent with the objectives of the current study and definitions of IS that emphasize the self-reported tendency to focus on internal bodily sensations (Desmedt et al., 2022).

### 2.4 Non-interoceptive control measures

A critique of the IAcc measure derived from the HTT is that participants may be able to infer the number of heartbeats occurring at each interval using prior knowledge of their typical heartrate and an estimation of elapsed time (Ring and Brener, 1996). Accordingly, IAcc scores could be high (participants appear to be accurate) if time estimation ability and heart rate knowledge were high even in the absence of actual interoceptive ability (Dunn et al., 2010). Prior work has also reported associations between IAcc scores and resting heart rate (Zamariola et al., 2018) as well as BMI (Robinson et al., 2021). Therefore, we obtained these non-interoceptive control measures to determine whether they were related to IAcc scores. The rationale for this analysis was that if these other variables were significantly correlated with IAcc score (e.g., individuals with higher IAcc also had higher time estimation ability and more accurate knowledge of resting heart rate), it was possible that these other control variables (e.g., time estimation ability and knowledge of heart rate) were responsible for higher IAcc instead of interoceptive ability.

#### 2.4.1 Resting heart rate and heart rate knowledge accuracy

Participants first reported an estimate of their typical resting heart rate (beats per minute). Resting heart rate was subsequently measured by the experimenter using palpation of the radial artery on the wrist. From these data, a *heartrate knowledge accuracy score* was calculated using the following formula:


$$H\,e\,a\,r\,t\,R\,a\,t\,e\,K\,n\,o\,w\,l\,e\,d\,g\,e\,A\,c\,c\,u\,r\,a\,c\,y=1-\left(\frac{\left|A\,c\,t\,u\,a\,l\,h\,e\,a\,r\,t\,r\,a\,t\,e-R\,e\,p\,o\,r\,t\,e\,d\,h\,e\,a\,r\,t\,r\,a\,t\,e\right|}{A\,c\,t\,u\,a\,l\,h\,e\,a\,r\,t\,r\,a\,t\,e}\right)$$

#### 2.4.2 Time estimation accuracy

Participants also completed a time estimation task (Ainley et al., 2014; Dunn et al., 2010). Similar to the HTT, the time estimation task had 6-time intervals (19s x2, 37s x2, 49s x2) presented in a pseudorandom order with auditory beeps indicating the start and end of each interval. Participants were instructed to silently count the number of seconds that occurred from the time they heard the first tone until they heard the second tone. Participants were told not to use a clock and to keep their eyes closed. The number of seconds reported by the participant were compared to the actual elapsed time to derive an index of *time estimation accuracy*:


$$T\,i\,m\,e\,E\,s\,t\,i\,m\,a\,t\,i\,o\,n\,A\,c\,c\,u\,r\,a\,c\,y=\frac{1}{6}\,\sum \left(1-\frac{\left|A\,c\,t\,u\,a\,l\,n\,u\,m\,b\,e\,r\,o\,f\,s\,e\,c\,o\,n\,d\,s-R\,e\,p\,o\,r\,t\,e\,d\,n\,u\,m\,b\,e\,r\,o\,f\,s\,e\,c\,o\,n\,d\,s\right|}{A\,c\,t\,u\,a\,l\,n\,u\,m\,b\,e\,r\,o\,f\,s\,e\,c\,o\,n\,d\,s}\right)$$

Heart rate knowledge accuracy and time estimation accuracy scores range from 0 to 1, with higher scores representing greater accuracy.

#### 2.4.3 Body mass index

Participants’ height and weight were recorded, and BMI was calculated (using the formula: reported weight (lb) ÷ reported height (inches)^2^ * 703).

### 2.5 Data analysis

Distributions of demographic, clinical, and behavioral measures were examined. Analyses on measures with normal distributions used ANOVA and Cohen’s d, whereas analyses on measures with non-normal distributions used Kruskal-Wallis H tests and delta median absolute deviation (Δ_MAD_, see Supplement 2.1 for additional details). Primary analyses assessed group differences in interoceptive and non-interoceptive control measures (heart rate, heart rate knowledge accuracy, time estimation accuracy, BMI). Relationships between IAcc and non-interoceptive control measures were examined within each group separately using Spearman rank correlations. Pairwise *post-hoc* comparisons following omnibus group tests were corrected for multiple comparisons using false discovery rate (FDR) correction within each set of follow-up comparisons.

Exploratory Spearman rank correlations were conducted without adjustment for multiple comparisons to probe for relationships among interoceptive measures (IAcc, IS, confidence), and between these measures and disorder-specific symptom severity, within each group separately.

Supplemental analyses examined the effects of sex, age, presence/absence of DSM-5 comorbidities, and psychotropic medication usage on interoceptive measures (Supplement: 2.2, 2.3, 2.4).

## 3 Results

### 3.1 Interoceptive accuracy (IAcc)

**FIGURE 1:**
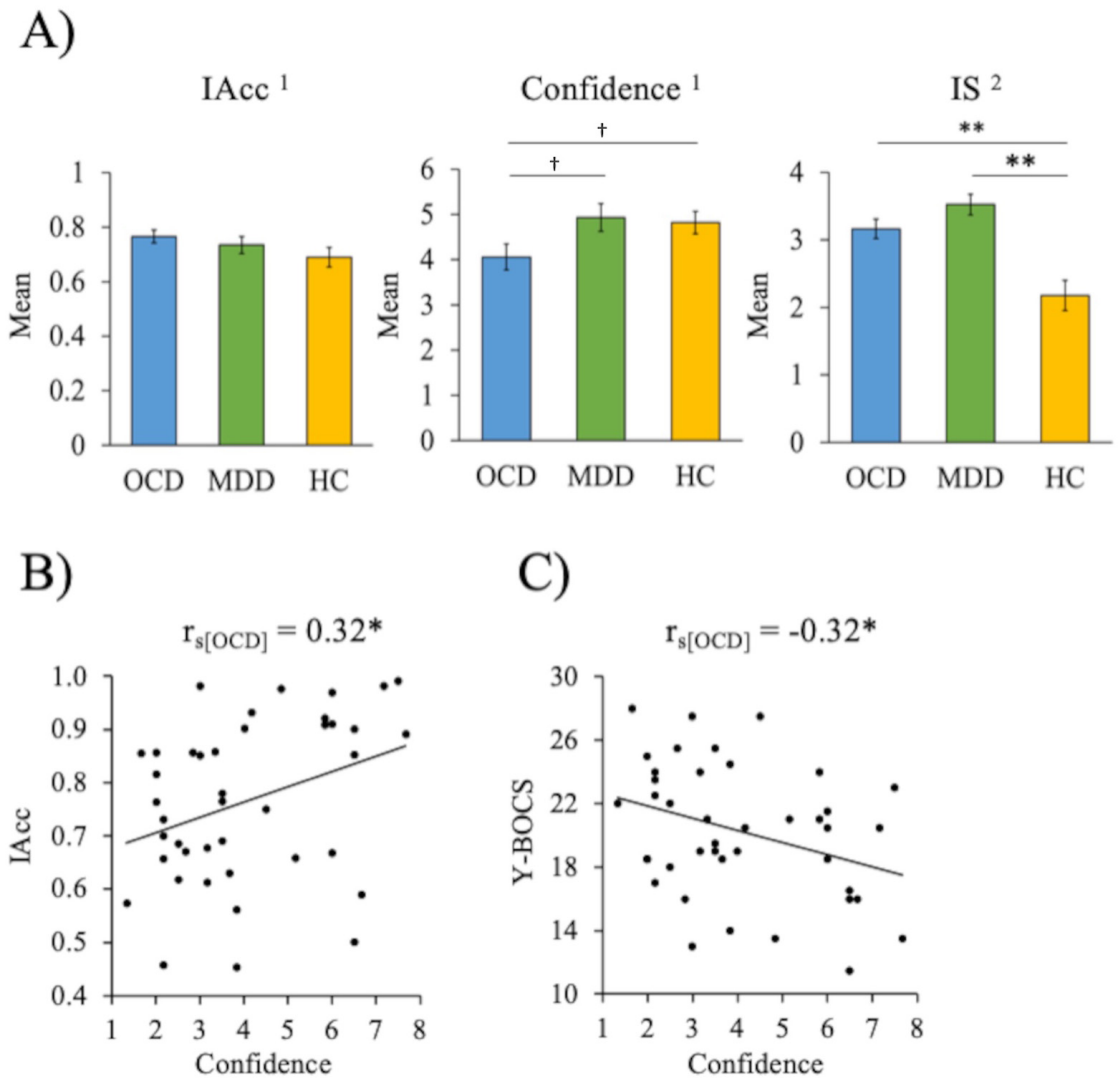
Group differences in interoceptive measures and correlations within the OCD group. **(A)** Bar graphs showing mean interoceptive accuracy (IAcc), confidence in interoceptive ability, and interoceptive sensibility (IS) in the OCD (*n* = 41), MDD (*n* = 38), and HC (*n* = 35) groups. **(B)** Scatterplot showing association between confidence in interoceptive ability and IAcc within OCD group (*n* = 41). **(C)** Scatterplot showing association between confidence in interoceptive ability and obsessive-compulsive symptom severity (as measured with the Yale-Brown Obsessive-Compulsive Scale, Y-BOCS) within OCD group (*n* = 41). Error bars reflect standard error of the mean. R-values from exploratory Spearman rank correlational analyses are reported with unadjusted *p*-values. IAcc, interoceptive accuracy (range: 0 to 1); Confidence = mean confidence rating of interoceptive ability reported during the HTT (range: 1 [not at all confident] to 9 [completely confident]); IS, Interoceptive Sensibility measured using the average score of the four questions from the Noticing subscale of the Multidimensional Assessment of Interoceptive Awareness (MAIA) self-report (range: 0 [never] to 5 [always]); Y-BOCS, Yale-Brown Obsessive-Compulsive Scale (range: 0 to 40). ^1^Assumption of normality not assumed. Statistical tests were performed using non-parametric Kruskal-Wallis H tests followed by *post-hoc* Dunn tests. ^2^Assumption of homogeneity of variance not assumed. Degrees of freedom were adjusted using Satterthwaite’s approximation. ^†^FDR-adjusted *p* = 0.06, **FDR-adjusted *p* < 0.001, *p < 0.05 (unadjusted).

Table 1 shows results from group comparisons and Figure 1A shows group means from the three interoceptive variables. Groups did not differ significantly in age, sex, or any of the non-interoceptive control measures (resting heart rate, heart rate knowledge accuracy, time estimation accuracy, or BMI; all *p* > 0.05).

**TABLE 1:** Demographic, clinical, and behavioral data.

Measure	OCD (*n* = 41) *Mean* ± *SD*	MDD (*n* = 35) *Mean* ± *SD*	HC (*n* = 38) *Mean* ± *SD*	Group comparisons
Demographics				
Sex assigned at birth (M/F, %M)	13/28, 32%	15/23, 39%	12/23, 34%	*n.s.*
Age (years)^1^	27.46 ± 8.29	29.66 ± 11.72	29.37 ± 9.66	*n.s.*
Clinical Measures				
Y-BOCS total score	20.24 ± 4.17	1.83 ± 3.59	–	–
IDS total score	8.63 ± 5.44	26.05 ± 6.44	–	–
Interoceptive measures				
Interoceptive accuracy (IAcc)^1,a^	0.77 ± 0.15	0.73 ± 0.19	0.69 ± 0.21	*n.s.*
Confidence in interoceptive ability^1,b^	4.06 ± 1.82	4.93 ± 1.88	4.82 ± 1.47	OCD < HC, Δ_*MAD*_ = 1.27^†^ OCD < MDD, Δ_*MAD*_ = 0.71^†^
Interoceptive sensibility (IS)^2,c^	3.16 ± 0.93	3.53 ± 0.94	2.18 ± 1.32	OCD > HC, *d* = 0.88** MDD > HC, *d* = 1.18**
Non-interoceptive control measures				
Body mass index^1^	24.53 ± 4.71	25.05 ± 5.18	24.95 ± 4.43	*n.s.*
Resting heart rate (bpm)	75.63 ± 10.64	75.66 ± 11.67	71.94 ± 7.97	*n.s.*
Heart rate knowledge accuracy^1^	0.87 ± 0.13	0.89 ± 0.10	0.88 ± 0.08	*n.s.*
Time estimation accuracy^1^	0.80 ± 0.20	0.76 ± 0.18	0.81 ± 0.12	*n.s.*

Effect sizes using Cohen’s d as a parametric measure of effect size and delta median absolute deviations (Δ_MAD_) as a non-parametric measure of effect size are presented for all significant pairwise comparisons. M, male; F, female; Y-BOCS, Yale-Brown Obsessive-Compulsive Scale (range: 0 to 40); IDS, Inventory of Depressive Symptomatology (range: 0 to 84); Δ_MAD_ = delta median absolute deviation; *d* = Cohen’s d.

^1^Assumption of normality not assumed. Statistical tests were performed using non-parametric Kruskal-Wallis H tests followed by *post-hoc* Dunn tests.

^2^Assumption of homogeneity of variance not assumed. Degrees of freedom were adjusted using Satterthwaite’s approximation.

^a^Interoceptive Accuracy assessed using the Heartbeat Tracking Task (HTT) (range: 0 to 1).

^b^Confidence in Interoceptive Ability is the mean confidence rating reported during the HTT (range: 1 [not at all confident] to 9 [completely confident]).

^c^Interoceptive Sensibility measured using the average score of the four questions from the Noticing subscale of the Multidimensional Assessment of Interoceptive Awareness (MAIA) self-report (range: 0 [never] to 5 [always]).

† FDR-adjusted *p* = 0.06, **FDR-adjusted *p* < 0.001.

For IAcc, there were no significant differences between the OCD, MDD, and HC groups (*p* > 0.05), which contrasts with results from prior studies (see Supplementary Table 3). Within the MDD group, greater IAcc was associated with greater resting heart rate (r_*s*_ = 0.36, *p* = 0.03), though this effect did not survive FDR correction (FDR-adjusted *p* = 0.1). No other significant correlations were observed between IAcc and the non-interoceptive control measures (all *p* > 0.05), indicating that interoceptive accuracy in this study could not be explained by performance on these non-interoceptive variables.

In order to determine whether the present study was underpowered to detect patient vs. control IAcc differences based on findings from the literature (Supplementary Table 3), a retrospective power analysis was conducted using the average effect size reported by prior studies that found significant IAcc differences in OCD patients (*n* = 2 studies; *d* = 0.86) and MDD patients (*n* = 4 studies; *d* = 0.65) compared to HCs (Supplementary Table 3). Results indicate the present study had 96% power to detect differences at α = 0.05 (two-tailed) between OCD and HC and 78% power to detect differences between MDD and HC.

### 3.2 Confidence in interoceptive ability

For confidence in interoceptive ability, the omnibus Kruskal-Wallis test indicated a significant group effect (*H*_2_ = 6.08, *p* = 0.048). Follow-up Dunn’s tests found lower confidence in the OCD group compared to the MDD (*p* = 0.029, *Δ_*MAD*_* = 0.71) and HC groups (*p* = 0.041, *Δ_*MAD*_* = 1.27); following FDR correction, both effects were observed at trend level (both FDR-adjusted *p* = 0.06). There was no significant difference between MDD and HC groups (*p* > 0.05).

### 3.3 Interoceptive sensibility (IS)

For IS, there was a significant group difference (*F*_2,70.3_ = 12.34, *p* < 0.001) such that MDD (*t*_61.1_ = 4.98, FDR-adjusted *p* < 0.001, *d* = 1.18) and OCD (*t*_59.8_ = 3.70, FDR-adjusted *p* < 0.001, *d* = 0.88) groups had higher IS than HCs but did not differ from each other (*p* > 0.05).

### 3.4 Associations among interoceptive measures

Exploratory analyses examining associations between the different interoceptive measures (IAcc, confidence, IS), found greater IAcc was associated with greater confidence in interoceptive ability (r_*s*_ = 0.32, *p* = 0.04, Figure 1B), but lower IS (r_*s*_ = −0.32, *p* = 0.04), in the OCD group. By contrast, within the MDD group, greater confidence was nominally associated with greater IS (r_*s*_ = 0.30, *p* = 0.07). In HCs, greater IAcc was nominally associated with lower IS (r_*s*_ = −0.33, *p* = 0.05), similar to the pattern found in the OCD group. No other associations were observed between interoceptive measures (all *p* > 0.05).

### 3.5 Associations between interoceptive measures and symptom severity

Exploratory analyses examining associations between interoceptive measures and symptom severity showed an association between greater obsessive-compulsive symptom severity (Y-BOCS total score) and lower confidence in the OCD group (*r_*s*_* = −0.32, *p* = 0.04, Figure 1C). No other associations with symptom severity were observed.

## 4 Discussion

This study provides a direct comparison of IAcc, confidence in interoceptive ability, and IS between patients with OCD, patients with MDD, and HCs using a validated heartbeat tracking task. Contrary to prior research (Demartini et al., 2021; Furman et al., 2013; Karanassios et al., 2021; Schultchen et al., 2019; Yoris et al., 2017), groups did not differ in IAcc. Based on effect sizes from published studies, our study had sufficient power to detect IAcc differences between OCD patients and controls. However, given that we only identified two previous studies, both with small sample sizes (Demartini et al., 2021; Schultchen et al., 2019), it is clear that future research using larger samples will be needed to resolve inconsistencies. Our study was slightly underpowered for the investigation of MDD; the null results found here are consistent with those from a recent study (Schulz et al., 2022), which reported no significant IAcc differences between relatively small samples of MDD (*n* = 24) and controls (*n* = 22).

Despite similar IAcc, OCD patients showed a pattern of lower confidence in their ability to correctly detect bodily (specifically heart) sensations relative to HCs and MDD patients, although pairwise effects were attenuated after FDR correction. Lower confidence was also associated with more severe obsessive-compulsive symptoms. These findings align with prior research reporting reduced interoceptive confidence in OCD patients compared to HCs (Yoris et al., 2017). A meta-analysis comparing OCD patients to HCs on actual and perceived accuracy across a variety of cognitive tasks (e.g., memory recall) demonstrated that OCD patients are under-confident in their cognitive abilities relative to their actual performance (Dar et al., 2022).

Exploratory analyses revealed that, within the OCD patient group, individuals with higher IAcc also had more confidence in their interoceptive ability, displaying a preserved correspondence between actual and self-reported accuracy during cardiac interoception that was not observed in MDD or HCs. This contrasts with findings from Yoris et al. (2017), who reported no group-level association between IAcc and confidence in OCD using a different cardiac interoceptive task in which participants tapped to indicate each perceived heartbeat, rather than silently counting heartbeats. Neither study indexed interoceptive awareness using within-participant correlations (e.g., correspondence between actual and self-reported accuracy on each trial), as proposed by Garfinkel et al. (2015). In the current study, this was not feasible due to low variability of confidence ratings over the six HTT trials. Future studies with more trials and confidence-rating variability are needed to directly probe interoceptive awareness in OCD.

Both OCD and MDD groups exhibited elevated IS in the form of noticing bodily sensations compared to HCs, which did not differ between OCD and MDD patients. This suggests that a heightened self-reported tendency to notice bodily sensations may be a shared feature of OCD and MDD, consistent with a prior study from our group comparing HCs to OCD in independent samples from those examined here (Eng et al., 2020), as well as studies comparing IS between MDD and HCs (Karanassios et al., 2021; Zhou et al., 2024). While the present study focused on the tendency to be aware of bodily sensations, this subscale represents only one dimension of IS, which is a multifaceted construct including not only the tendency to notice sensation as measured here, but also the tendency to attend to, interpret, and regulate responses to bodily signals. Indeed, prior work has found that both patients with OCD (Eng et al., 2020) and those with MDD (Dunne et al., 2021; Zhou et al., 2024) exhibit a greater self-reported tendency to distract from, worry about, and distrust bodily sensations when separately compared to HCs. Future research could build on these findings by comparing attentional and regulatory aspects of interoception in OCD and MDD.

Limitations of this study should be noted. Consistent with prior work, this study measured IAcc in the context of cardiac interoception using the HTT, however, performance on this task can be influenced by non-interoceptive factors such as prior knowledge of resting heart rate and time estimation ability (Zamariola et al., 2018). Although groups did not differ on these control measures and IAcc was not consistently associated with these variables within groups, future studies using alternative cardiac interoception tasks, such as the heartbeat discrimination task (Hickman et al., 2020) or phase-adjustment paradigm (Plans et al., 2021) may be needed to replicate the null findings reported here.

Additionally, while the current study focused on clinically representative OCD and MDD samples, the presence of psychiatric comorbidities complicates attribution of the observed effects to OCD or MDD diagnoses alone. Furthermore, sex differences have been reported in interoception (Grabauskaite et al., 2017; Prentice and Murphy, 2022; Spooner et al., 2024), and supplemental analyses showed that the lower confidence observed in OCD relative to MDD and HCs was attenuated when covarying for sex (Supplement: Section 2.2–2.3). Future studies examining larger samples of OCD and MDD stratified by sex, comorbidity, and medication status may provide further insights into interoception in OCD and MDD.

Despite these limitations, results provide preliminary evidence of shared and potentially disorder-specific alterations of interoception in OCD and MDD, with potential implications for treatment. Future work should clarify whether interventions such as mindfulness or interoceptive exposure-based approaches could be used to modify interoceptive processes in OCD and MDD (Khalsa et al., 2018).

## Data Availability

The raw data supporting the conclusions of this article will be made available by the authors, without undue reservation.
