# Supplementary material for: Interoception in obsessive-compulsive and major depressive disorders
Source: Front Psychol. 2026 Aug 5;17:1856945. doi: 10.3389/fpsyg.2026.1856945 (PMC13485727; doi:10.3389/fpsyg.2026.1856945)
Supplement: Supplementary file 1 [file Data_Sheet_1.pdf]

Table of Contents

1 Methods and Materials 2
1.1 Participants 2
1.2 The Heartbeat Tracking Task 2
2 Data Analyses and Results 2
2.1 Distributions and Group Comparisons 2
2.2 Relationship Between Interoceptive and Demographic Measures 3
2.3 Group Differences in Confidence and Interoceptive Sensibility While Controlling for the Effects of Sex 3
2.4 Patient Group Differences in Confidence While Controlling for SSRI Usage and Current DSM-5 Comorbidity 4
3 Supplementary Tables 5
3.1 Table S1. 5
3.2 Table S2 6
3.3 Table S3 7
4 References 8

1 Methods and Materials

1.1 Participants

Within the OCD group (n = 41), 31 individuals (76%) met criteria for at least one current DSM-5 comorbidity and 30 individuals (73%) were currently taking at least one psychotropic medication. Within the MDD group (n = 38), 32 individuals (84%) met criteria for at least one current DSM-5 comorbidity and 23 individuals (61%) were currently taking at least one psychotropic medication. Refer to Table S2 and Table S3 for information on types of comorbid diagnoses and psychotropic medications in patient samples.

Chi-Square tests for independence revealed that OCD and MDD groups did not differ significantly in the proportion of individuals with one or more current DSM-5 comorbidities (presence/absence) or in the proportion of individuals currently taking one or more psychotropic medications (presence/absence, all $p > 0.05$).

1.2 The Heartbeat Tracking Task

Participants were seated in a chair in a quiet room and instructed to keep their legs uncrossed and their feet flat on the floor. A pulse transducer (BN-PULSE-XDCR Pulse Transducer, BioNomadix) was placed on the right index finger. The right hand was placed on a table and the right index finger and transducer complex was loosely taped to the table to minimize movement. Two auditory tones indicated the start and end of each count interval. Participants were instructed to close their eyes and silently count the number of times they felt their heartbeat from the time they heard the first tone until they heard the second tone. Participants were additionally instructed to try to feel the sensation of their heartbeat coming from within their body and without measuring their pulse (participants were not permitted to use their fingers to feel their pulse). Photoplethysmography (PPG) recorded cardiac waveforms using BIOPAC Systems Inc. MP system with AcqKnowledge 5.0 software.

2 Data Analyses and Results

2.1 Distributions and Group Comparisons

The distributions of IAcc and confidence in interoceptive ability differed significantly from normal in at least one of the three groups ($p < 0.05$), thus group differences were analyzed using non-parametric Kruskal-Wallis H tests, followed by Dunn tests for pairwise comparisons. Group differences in IS were evaluated using a one-way ANOVA, followed by independent sample t-tests for pairwise comparisons (degrees of freedom adjusted using Satterthwaite's approximation). Effect sizes are reported as Cohen's d and delta median absolute deviation (Δ_{MAD}) (Ricca and Blaine, 2020) for significant results from parametric and non-parametric comparisons, respectively.

The distributions of resting heart rate did not differ significantly from normal in the OCD, MDD, or HC groups (all $p > 0.05$), however, at least one of the groups had a distribution that differed significantly from normal for all remaining non-interoceptive control measures (heart rate knowledge accuracy, time estimation accuracy, and BMI, all $p < 0.05$). Therefore, group differences in resting heart rate were evaluated using a one-way ANOVA, while group differences in the remaining measures were evaluated by Kruskal-Wallis H tests.

2.2 Relationship Between Interoceptive and Demographic Measures

Spearman ranks correlational analyses examined the relationship between age and interoceptive measures (IAcc, confidence, IS) within each group separately. Age was not significantly related to any of the interoceptive measures (all $p > 0.05$).

Previous studies have reported a significant effect of sex assigned at birth on IAcc (Prentice and Murphy, 2022), confidence in interoceptive ability (Spooner et al., 2024), and IS (Grabauskaite et al., 2017). Therefore, the effects of sex assigned at birth on these interoceptive measures were evaluated within each group using Mann-Whitney U tests. Within the OCD group, males (Mdn = 5.83, n = 13) reported significantly greater confidence in their interoceptive ability compared to females (Mdn = 3.33, n = 28), U = 93.0, z = -2.50, p = 0.01, FDR-adjusted p = 0.04, Δ_{MAD} = 1.89. Within the HC group, males (n = 12) displayed greater IAcc (U = 72.0, z = -2.29, p = 0.02, FDR-adjusted p = 0.05, Δ_{MAD} = 1.25), greater confidence (U = 82.0, z = -1.95, p = 0.05, FDR-adjusted p = 0.05, Δ_{MAD} = 1.42), and lower IS (U = 77.0, z = 2.13, p = 0.03, FDR-adjusted p = 0.05, Δ_{MAD} = 2.05) than females (n = 23). There were no significant effects of sex assigned at birth on the interoceptive measures the MDD group (all $p > 0.05$).

2.3 Group Differences in Confidence and Interoceptive Sensibility While Controlling for the Effects of Sex

Because there were significant relationships between sex assigned at birth and confidence in interoceptive ability, as well as IS, we repeated our analyses examining group differences in IS and confidence while statistically adjusting for variance associated with sex by specifying sex as a covariate in a Quade's ANCOVA (Quade, 1967).

Differences in IS between the OCD, MDD, and HC groups remained significant when covarying for sex (F (2,111) = 13.23, $p < 0.001$).

Differences in confidence between the OCD, MDD, and HC groups became trend-level significant when covarying for sex (F (2,111) = 2.98, p = 0.06). Follow up Kruskal-Wallis H tests compared confidence between the three groups separately for males (n = 40) and females (n = 74). Among females, individuals with OCD had significantly lower confidence than those with MDD (p = 0.003, FDR-adjusted p = 0.009, Δ_{MAD} = 1.15) and HCs (p = 0.025, FDR adjusted p = 0.04, Δ_{MAD} = 1.07), H (2) = 10.04, p = 0.007, consistent with the effect in the full sample

reported in the main manuscript. Among males, confidence levels did not significantly differ between the OCD, MDD and HC groups (H (2) = 0.29, $p > 0.05$).

2.4 Patient Group Differences in Confidence While Controlling for SSRI Usage and Current DSM-5 Comorbidity

Because previous work has implicated selective serotonin reuptake inhibitor (SSRI) use in interoceptive measures (Burrows et al., 2022), and given the high rate of psychiatric comorbidity in the clinical groups, we tested whether the confidence difference between OCD and MDD patients remained significant after accounting for these variables. To do so, we conducted two Quade's ANCOVAs (Quade, 1967), one adjusting for current SSRI use (presence/absence), and one adjusting for current DSM-5 comorbidities (presence/absence).

These sensitivity analyses were restricted to clinical participants because HCs were free of psychotropic medication and DSM-5 comorbidities by design, making these variables confounded with clinical status in the full sample.

Confidence remained significantly lower in OCD than MDD after rank-adjusting for current SSRI use, $F(1,77) = 7.02$, $p = 0.01$. Similarly, confidence remained significantly lower in OCD than MDD after rank-adjusting for current DSM-5 comorbidity status, $F(1,77) = 4.76$, $p = 0.03$.

3 Supplementary Tables

3.1 Table S1.

DSM-5 comorbidities in OCD and MDD patients.

	**OCD Group (n = 41)**	**MDD Group (n = 38)**
**Comorbidity Status**	n (%)	n (%)
No DSM-5 Comorbidity	10 (24%)	6 (16%)
At least 1 current DSM-5 Comorbidity	31 (76%)	32 (84%)
**Comorbid Diagnosis**	n	n
Attention Deficit Hyperactivity Disorder	10	12
Body Dysmorphic Disorder	10	7
Generalized Anxiety Disorder	9	11
Alcohol Use Disorder (Mild or Moderate)	7	2
Social Anxiety Disorder	5	10
Excoriation	4	2
Panic Disorder	4	2
Somatic Symptom Disorder	4	2
Substance Use Disorder (Mild or Moderate)	4	6
Agoraphobia	2	3
Post Traumatic Stress Disorder	1	7
Tic Disorders	2	0
Bulimia Nervosa (Moderate)	1	0
Hoarding disorder	1	0
Binge-Eating Disorder (Mild)	0	4

Note. Individuals in the OCD group met criteria for a current diagnosis of OCD without a comorbid current diagnosis of MDD. Individuals in the MDD group met criteria for a current diagnosis of MDD without a comorbid current diagnosis of OCD. Tic Disorders includes Tourette's disorder and Persistent (Chronic) Motor or Vocal Tic Disorder

3.2 Table S2

Psychotropic medication information for OCD and MDD patients.

		OCD (n = 41)	MDD (n = 38)
	**Medication Status**	*n*	*n*
	Unmedicated	11 (27%)	15 (39%)
	Medicated	30 (73%)	23 (61%)
**Medication Class**	**Medication**	*n*	*n*
SSRIs	Escitalopram, Fluoxetine, Fluvoxamine, Paroxetine, Sertraline, Vilazodone Hydrochloride	25	13
SNRIs	Desvenlafaxine, Venlafaxine	1	2
Atypical antidepressants	Bupropion Hydrochloride, Trazodone, Vortioxetine, Mirtazapine	8	7
Stimulants	Amphetamine salts, Dexmethylphenidate, Methylphenidate	7	5
Triptans	Almotriptan, Rizatriptan, Sumatriptan	5	1
Benzodiazepines	Alprazolam, Clonazepam, Lorazepam, Diazepam	4	5
Non-benzodiazepine anxiolytics	Hydroxyzine, Buspirone, Propranolol	3	5
Anticonvulsants	Gabapentin, Lamotrigine, Topiramate	2	4
Atypical antipsychotics	Aripiprazole, Lurasidone Hydrochloride, Olanzapine, Quetiapine, Brexpiprazole	2	4
Hypnotics	Eszopiclone, Zolpidem	1	3
Other	Medical marijuana, L-methylfolate, Botox, Tramadol	2	2

Note. SSRI = selective serotonin reuptake inhibitor, SNRI = serotonin–norepinephrine reuptake inhibitor

3.3 Table S3

Studies used for retrospective power analysis.

Authors (year)	Sample	Heartbeat Tracking Task	Design	Comparison	Finding	Effect Size
Terhaar et al., 2012 (11)	MDD, n=15<br>HC, n=15	3 intervals (each presented once: 35s, 25s, 45s)	Cross-sectional	One-tailed t-test	Lower IAcc in MDD (M=0.67, SD=0.19 †) vs. HC (M=0.81, SD=0.15 †).	0.82
Furman et al., 2013 (12)	MDD, n=25<br>HC, n=36	3 intervals (each presented once: 35s, 25s, 45s)	Cross-sectional	One-tailed t-test	Lower IAcc in MDD (M=0.55, SD=0.21) vs. HC (M=0.65, SD=0.20).	0.49
Schultchen et al., 2020 (13)	MDD, n=36<br>HC, n=36	4 intervals (each presented once: 25s, 35s, 45s, 60s)	Cross-sectional	Two-tailed t-test	Lower IAcc in MDD (M=0.62, SD=0.21) vs. HC (M=0.77, SD=0.17).	0.79
Karanassios et al., 2021 (14)	MDD, n=60<br>HC, n=52	4 intervals (each presented once: 25s, 35s, 45s, 60s)	Longitudinal (pre-post CBT ± MBSR)	One-tailed t-test	Lower IAcc in MDD (M=0.63, SD=0.21) vs. HC (M=0.73, SD=0.19) at baseline.	0.50
Schultchen et al., 2019 (15)	OCD, n=26<br>HC, n=26	3 intervals (each presented once: 25s, 35s, 45s)	Longitudinal (pre-post CBT + exposure)	Repeated-measures ANOVA	Lower IAcc in OCD (M=0.60, SD=0.17) vs. HC (M=0.75, SD=0.17) at baseline.	0.88
Demartini et al., 2021 (16)	OCD, n=18<br>HC, n=18	3 intervals (each presented once: 25s, 35s, 45s)	Cross-sectional	Two-tailed t-test	Lower IAcc in OCD (M=0.41, SD=0.31) vs. HC (M=0.64, SD=0.24).	0.83

Note. Selected studies measured interoceptive accuracy (IAcc) using the Schandry Heartbeat Tracking Task (Schandry, 1981). Effect sizes calculated using reported means and standard deviations in G*Power (Version 3.1.9.6) (Faul et al., 2007). Abbreviations: CBT = cognitive behavioral therapy, MBSR = mindfulness-based stress reduction, OCD = obsessive-compulsive disorder, MDD = major depressive disorder, HC = healthy control.

† SD was not reported by the authors and was estimated using the reported SEM and sample size as SD = SEM * √n.

4 References

Burrows, K., DeVille, D.C., Cosgrove, K.T., Kuplicki, R.T., Tulsa, I., Paulus, M.P., Aupperle, R., Khalsa, S.S., Stewart, J.L., 2022. Impact of serotonergic medication on interoception in major depressive disorder. Biol Psychol 169, 108286. https://doi.org/10.1016/j.biopsycho.2022.108286

Faul, F., Erdfelder, E., Lang, A.-G., Buchner, A., 2007. G*Power 3: A flexible statistical power analysis program for the social, behavioral, and biomedical sciences. Behav. Res. Methods 39, 175–191. https://doi.org/10.3758/BF03193146

Grabauskaite, A., Baranauskas, M., Griskova-Bulanova, I., 2017. Interoception and gender: What aspects should we pay attention to? Conscious Cogn 48, 129–137. https://doi.org/10.1016/j.concog.2016.11.002

Prentice, F., Murphy, J., 2022. Sex differences in interoceptive accuracy: A meta-analysis. Neurosci Biobehav Rev 132, 497–518. https://doi.org/10.1016/j.neubiorev.2021.11.030

Quade, D., 1967. Rank Analysis of Covariance. J. Am. Stat. Assoc. 62, 1187–1200. https://doi.org/10.1080/01621459.1967.10500925

Ricca, B.P., Blaine, B.E., 2020. Brief Research Report: Notes on a Nonparametric Estimate of Effect Size. J. Exp. Educ. 90, 249–258. https://doi.org/10.1080/00220973.2020.1781752

Schandry, R., 1981. Heart beat perception and emotional experience. Psychophysiology 18, 483–488.

Spooner, R., Bird, J.M., Irigoras Izagirre, N., Clemente, R., Fernandez Fueyo, E., Budworth, G., Cocirla, D., Todd, J., Aspell, J., Leganes, M., Watling, D., Plans, D., Brewer, R., Murphy, J., 2024. Exploring sex differences in cardiac interoceptive accuracy using the phase adjustment task. Psychophysiology e14689. https://doi.org/10.1111/psyp.14689
